# Changes in Working Situations of Employed Long COVID Patients: Retrospective Study in Japanese Outpatient Clinic

**DOI:** 10.3390/jcm13133809

**Published:** 2024-06-28

**Authors:** Yui Matsuda, Yasue Sakurada, Yuki Otsuka, Kazuki Tokumasu, Yasuhiro Nakano, Naruhiko Sunada, Hiroyuki Honda, Toru Hasegawa, Ryosuke Takase, Daisuke Omura, Keigo Ueda, Fumio Otsuka

**Affiliations:** Department of General Medicine, Graduate School of Medicine, Dentistry and Pharmaceutical Sciences, Okayama University, 2-5-1 Shikata-Cho, Kita-Ku, Okayama 700-8558, Japan; phvw0350@okayama-u.ac.jp (Y.M.);

**Keywords:** employment, job retirement, leave of absence, long COVID, omicron variant, post-COVID-19 condition

## Abstract

**Purpose**: The present study aimed to uncover the impact of long COVID on the working situations of Japanese patients. **Methods**: Changes in the working situations of the patients who visited our long COVID clinic were evaluated from medical records for the aspects of physical status, quality of life (QOL), and mental conditions. **Results**: Of 846 long COVID patients who visited our clinic from February 2021 to December 2023, 545 employed patients aged between 18 and 65 years were included in this study. A total of 295 patients (54.1%) with long COVID (median age: 43 years, female: 55.6%) experienced changes in their working status. Those patients included 220 patients (40.4%) who took a leave of absence, 53 patients (9.7%) who retired, and 22 patients (4%) with reduced working hours. Most of the patients (93.2%) with changes in working conditions had mild disease severity in the acute phase of COVID-19. The majority of those patients with mild disease severity (58.8%) were infected in the Omicron-variant phase and included 65.3% of the female patients. The major symptoms in long COVID patients who had changes in their working situations were fatigue, insomnia, headache, and dyspnea. Scores indicating fatigue and QOL were worsened in long COVID patients who had changes in their working situations. In addition, 63.7% of the long COVID patients with changes in their working situations had decreases in their incomes. **Conclusions:** Changes in the working situation of long COVID patients who were employed had a negative impact on the maintenance of their QOL.

## 1. Introduction

Various variants with viral mutations have emerged since the beginning of the coronavirus disease 2019 (COVID-19) pandemic [1,2]. Approximately one-third of patients with COVID-19 are likely to experience prolonged illness following the acute phase of COVID-19 [3,4]. Various physical and mental symptoms due to prolonged illness, including fatigue and depression, have been recognized by many institutions, including the World Health Organization [5]. However, the detailed mechanisms underlying the development of the post-infectious sequalae, so-called long COVID, have yet to be clarified.

Long COVID is a prolonged illness that can develop after infection with the SARS-CoV-2 virus. The development of long COVID is usually not dependent on the severity of COVID-19 in the acute phase. Long COVID has been defined as symptoms that occur 2 to 3 months after the viral infection and last for more than 2 months [5]. The major symptoms of long COVID are general fatigue; so-called post-exertional malaise; accompanying headache, insomnia, continuous cough, and dyspnea; and some cases also have symptoms of cognitive impairment, mild fever, and muscle pain [6,7]. The mechanisms underlying the pathophysiological aspects of long COVID include viral persistence and reactivation of latent viruses, immune dysregulation and autoimmunity, dysregulation of microbiomes, abnormalities of the autonomous nerve system, tissue damage due to microclots, and endocrine dysfunctions [6,8,9].

The symptomatic characteristics of long COVID patients have gradually changed, and fatigue, headache, and insomnia became the main symptoms in patients during the Omicron-variant phase [10,11]. The symptoms of long COVID are partly different in a variant-dependent manner, although the reason has yet to be elucidated. In our previous study, it was shown that the number of long COVID patients with fatigue-related symptoms has been gradually increasing during the Omicron phase regardless of the severity of the acute phase of infection [12]. On the other hand, the frequencies of dysosmia, dysgeusia, and hair loss have been decreasing. In addition, the trends of long COVID symptoms in the Omicron period have made a clinical diagnosis more complicated because of the necessity for differentiating it from various mimicking diseases [13].

However, since the major symptom of long COVID is general fatigue, the influence of fatigue symptoms on working and/or employed conditions, including the types of jobs and the severity and length of working, might be unavoidable. After COVID-19 illness, some patients suffer from physical stress and mental stress with no prospect of symptomatic recovery [14]. Some patients feel guilty about their inability to perform well at work due to their symptoms and consider quitting work [15]. Also, several researchers have indicated that long COVID has resulted in an average reduction in working time and a decrease in income [16,17].

As for long COVID cases in Japan, when an insured person is unable to work due to illness or injury, they can receive a sickness and injury allowance for a certain period. In general, the allowance is approximately 80% of the insured person’s pre-illness income and the allowance period is a maximum of 1 year and 6 months in Japan. This study aims to reveal the impact of long COVID on the working hours of patients who visited our outpatient clinic in Japan.

## 2. Subjects and Methods

### 2.1. Study Design and Clinical Assessment of Long COVID

As a retrospective study, the medical records of all patients aged 10 years or older who visited the COVID-19 aftercare clinic (CAC; General Medicine Department) at Okayama University Hospital during the period from 15 February 2021 until 31 December 2023 were carefully reviewed. The CAC was established in the western part of Japan, and we have been treating long COVID patients who visited the CAC for more than 3 years, since February 2021 [12,18,19]. Long COVID has been defined as symptoms persisting for longer than one month after COVID-19 illness [20]. The medical records were reviewed during the period from 6 February to 19 March in 2023 and information on the patients’ age, gender, body mass index (BMI), smoking habit, severity in the acute phase of COVID-19, duration between infection and first visit to the CAC, vaccination history for COVID-19, date of onset of the acute phase of COVID-19, working status, and clinical symptoms of long COVID was collected. The definition of the severity of COVID-19 in the acute phase was based on the criteria proposed by the Ministry of Health, Labour and Welfare in Japan [21]. All of the patients received detailed face-to-face interviews with physicians for the assessment of symptoms.

### 2.2. Inclusion and Exclusion Criteria

Of the 866 patients who visited the CAC during the study period, 11 patients who first visited our clinic less than one month after the onset of COVID-19 and 9 patients who had unconfirmed COVID-19 or no clear complaint due to infection were excluded from the analysis in this study. In addition, 154 patients under 18 years or over 65 years of age and 147 patients who were not employed or not working were excluded. The remaining 545 long COVID patients were included in the analysis in this study.

### 2.3. Impact of Long COVID on Working Situations

For the treatment of the patients, a detailed medical interview and examination was carried out by 22 doctors in the outpatient department of the hospital. In this study, information on employment was obtained from these medical records by two investigators (Y.M. and Y.S.) and the changes in working situations were categorized as follows: “reduced working hours”, meaning shortened working hours or some kind of work restriction for a duration of more than one month; “leave of absence”, meaning absence from work for more than one month; and “retired from working”, indicating resignation from the employed job with or without leave of absence or shortened working hours. Information on changes in income was obtained from a questionnaire survey.

### 2.4. Setting for the Periods of COVID-19 Viral Variants

In accordance with our earlier report [12], we separated the COVID-19 onset periods into three categories: “Preceding phase”, which indicates the time frame from the ancestral variant to the Alpha variant prior to 18 July 2021; “Delta-dominant phase”, which includes cases that occurred from 19 July 2021 to 31 December 2021, when the Delta variant was dominant following the Alpha-variant phase; and “Omicron-dominant phase”, which indicates the period since the first appearance of the Omicron variant on 1 January 2022 in the Okayama district.

### 2.5. Evaluation of Quality of Life and Mental Condition

Patients were requested to fill in a questionnaire using the Japanese version of the fatigue assessment scale (FAS) [22] and the Japanese version of the Euro QOL five dimensions five levels (EQ-5D-5L) to evaluate their fatigue status and the level of their quality of life (QOL) [23]. As for the FAS score, we verified the structural validity and internal consistency reliability of the Japanese version of the FAS earlier, indicating that the Japanese version of the FAS is useful for assessing general fatigue in patients with long COVID in Japan [24]. In the EQ-5D-5L assessment, a score of ‘0’ means the worst QOL and a score of ‘1’ means the best QOL. For the EQ-5D VAS, a score of ‘0’ means the worst QOL and a score of ‘100’ means the best QOL. The self-rating depression scale (SDS) questionnaire was used to estimate mental health status, including depression [25,26].

### 2.6. Statistical Analysis

We used Stata SE, version 18, statistical software (StataCorp., College Station, TX, USA) for all analyses. The Mann–Whitney U test and Student’s *t*-test were used for variables that were not normally distributed and variables that were normally distributed, respectively, and Pearson’s chi-square test was used for categorical variables. Multivariate logistic analysis was additionally performed for calculating odds ratios. * *p* < 0.05 and ** *p* < 0.01 were defined as significantly different.

### 2.7. Ethical Approval

The study protocol was approved by the Okayama University Hospital Ethics Committee (No. 2105-030) and complied with the Declaration of Helsinki. Information about the study was shown on our hospital’s website. The opportunity to opt out of the study was given to patients who so wished. Informed consent was not required from the patients as the data in this study underwent anonymization procedures.

## 3. Results

First, it was shown that the working situations of 295 (54.1%) of the 545 patients were affected to some degree by long COVID, as can be seen in Table 1, while the working conditions of the remaining 250 patients (45.9%) were not obviously affected. The median ages of the long COVID patients were 43 years in both the groups with and without changes in working situations (Table 1). The proportion of female patients in the group with changes in working situations (164 female patients, 55.6%) was significantly larger than that of male patients (Table 1). Median body mass index (BMI) was approximately 23 and was not different between the two groups. The proportion of patients with a smoking habit was also not significantly different between the two groups (Table 1).

As shown in Table 1, the proportion of long COVID patients with changes in their working situations for whom the initial infection was mild (93.2%) was significantly higher than the proportion of patients with moderate/severe infections (6.8%). Among the patients with mild severity in the acute phase, 56.1% of the patients had changed working situations, while 36.4% of the moderate/severe infection cases had changed working situations (Table 1). Among the long COVID patients who had changes in their working situations, the proportion of patients who visited the CAC within 90 days from the primary infection was significantly larger than the proportion of patients who visited the CAC more than 90 days from the primary infection (54.2% vs. 45.8%). There was no difference in the COVID-19 vaccination status between the groups with and without changes in their working situations. Notably, the long COVID patients with changes in their working situations included a significantly higher percentage of patients with decreased incomes than the percentage of patients with decreased incomes among the long COVID patients without changes in their working situations (63.7% vs. 33.6%; Table 1). In addition, among the long COVID patients with decreased income, a large population of the patients (69.1%) were found to have changed working situations (Table 1).

**Figure 1:**
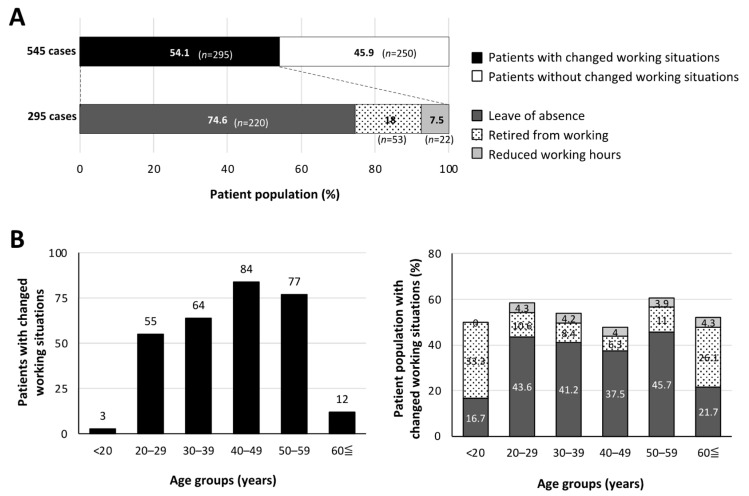
Long COVID patients whose working situations were affected. (**A**) Changes in working situations. (**B**) Age-dependent distribution of long COVID patients who had changes in working conditions and details of working status. Explanatory notes for bar graphs are the same for panels (**A**,**B**). “*n*” denotes the number of patients.

The long COVID patients who had changes in their working situation are shown in Figure 1. Of the long COVID patients with changes in their working situations, 220 patients (74.6%) took a leave of absence, 53 patients (18%) retired from their work, and 22 patients (7.5%) had reduced working hours (Figure 1A). The age range with the largest proportion of patients who had changes in their working situations was 40 to 49 years (84 patients, 28.5%) (Figure 1B, left panel). Approximately half of the patients in each age group had changes in their working conditions (Figure 1B, right panel). Notably, young patients (<20 years of age) and older patients (≥60 years of age) both included a higher percentage of patients who retired from work than the percentage of patients with reduced working hours (Figure 1B, right panel).

**Figure 2:**
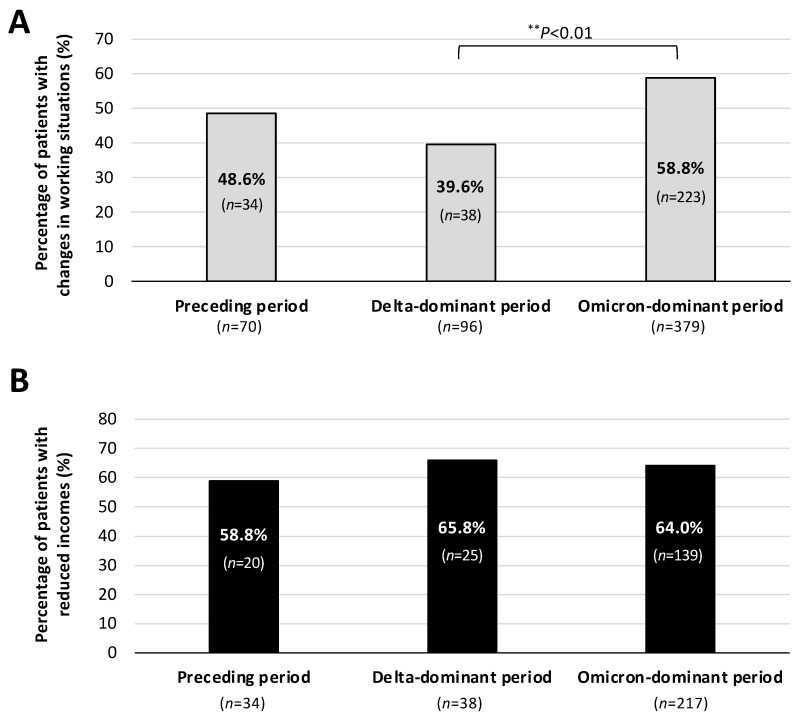
Long COVID patients with changes in working situations in each of the infection phases of COVID-19. (**A**) Patients with changes in working conditions in the Preceding, Delta-dominant, and Omicron-dominant periods. (**B**) Percentages of patients with reduced income. The *chi*-square test was performed; statistical significance between the indicated groups is shown as ** *p* < 0.01. “*n*” denotes the number of patients.

**Figure 3:**
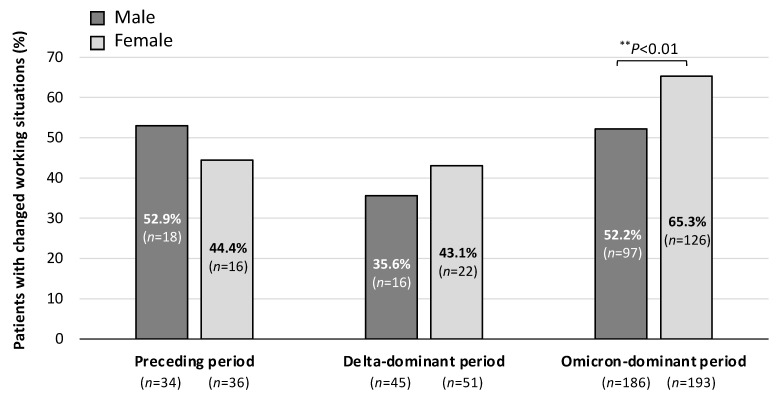
Gender-dependent percentages of long COVID patients with changes in working situations in the infection phases of COVID-19. Male and female patients with changes in working conditions in the Preceding, Delta-dominant, and Omicron-dominant periods are shown. The chi-square test was performed; statistical significance between the indicated groups is shown as ** *p* < 0.01. “*n*” denotes the number of patients.

Since the symptoms due to long COVID have been gradually altered depending on the viral variants [12], the main symptoms that affected patients’ working situations were examined. Figure 2 shows the percentage of long COVID patients with changes in their working situations in each of the periods of COVID-19 onset. As shown in Figure 2A, among the three periods, including the Preceding period, Delta-dominant period, and Omicron-dominant period, the percentage of long COVID patients with changes in their working situations was significantly higher in the Omicron-dominant period than in the Delta-dominant period (58.8% vs. 39.6%). Furthermore, as shown in Figure 2B, approximately 60% of the patients who had changes in their working situations had reduced incomes regardless of the COVID-19 period. The percentages of male and female long COVID patients who had changes in their working situations are shown in Figure 3. Among the patients infected in the Omicron-dominant period, the proportion of female patients with changes in their working situations was significantly higher than that of male patients (65.3% vs. 52.2%).

**Figure 4:**
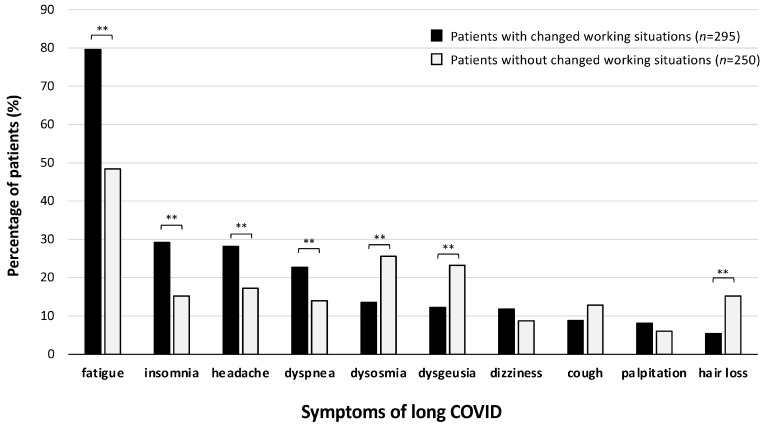
Percentages of patients with each long COVID symptom among the patients with and those without changes in working situations. The percentages of patients with major long COVID symptoms in the groups with and without changes in working conditions are shown. The *chi*-square test was performed; statistical significance between the indicated groups is shown as ** *p* < 0.01. “*n*” denotes the number of patients.

The number of patients with each long COVID symptom at the first CAC visit is shown in Figure 4. Fatigue was the most frequent symptom not only in patients with changes in their working situations but also in those without changes in their working situations. The percentage of patients experiencing fatigue was significantly higher in the group with changes in working situations than in the group without changes in working situations (79.7% vs. 48.4%). The percentages of patients with insomnia (29.2% vs. 15.2%), headache (28.1% vs. 17.2%), and dyspnea (22.7% vs. 14%) were significantly higher in patients with changes in their working situations than in those without changes in their working situations (Figure 4). On the other hand, the percentages of patients with dysosmia (25.6% vs. 13.6%), dysgeusia (23.2% vs. 12.2%), and hair loss (15.2% vs. 5.4%) were rather higher in patients without changes in their working situations. Based on the logistic analysis, the odds ratios of these symptoms for affecting working situations were as follows: 3.39 [95% Confidence Interval (CI): 2.28–5.04] for fatigue (** *p* < 0.01); 1.69 [95%CI: 1.07–2.66] for insomnia (* *p* < 0.05); 1.42 [95%CI: 0.90–2.25] for headache (*p* = 0.13); 1.51 [95%CI: 0.92–2.48] for dyspnea (*p* = 0.10); 0.93 [95%CI: 0.48–1.81] for dysosmia (*p* = 0.84); 0.61 [95%CI: 0.31–1.21] for dysgeusia (*p* = 0.15); 0.96 [95%CI: 0.52–1.78] for dizziness (*p* = 0.90); 0.61 [95%CI: 0.34–1.11] for cough (*p* = 0.11); 1.00 [95%CI: 0.48–2.08] for palpitation (*p* = 1.00); and 0.39 [95%CI: 0.20–0.75] for hair loss (** *p* < 0.01).

**Figure 5:**
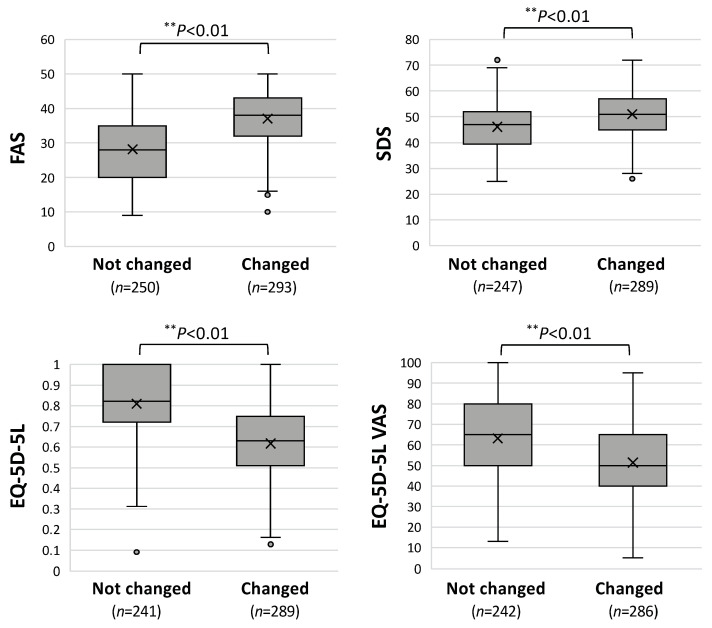
Comparison of self-assessed scales for fatigue, quality of life, and depression in long COVID patients with and without changes in working situations. FAS: fatigue assessment scale; EQ-5D-5L: Euro QOL 5-dimensions 5-levels score; VAS: visual analog scale; and SDS: self-rated depression scale. The box means interquartile range, the horizontal bar in the middle indicates the median, “x” indicates the mean, and the lower and upper horizontal bars outside show the minimum and maximum values within 1.5-fold of the interquartile ranges, respectively. Data for the FAS, EQ-5D-5L, and EQ-5D VAS were analyzed by the Mann–Whitney U test, and the SDS was analyzed by Student’s *t*-test; statistical significance between the indicated groups is shown as ** *p* < 0.01. “*n*” denotes the number of patients.

Finally, the self-rated scales regarding “fatigue”, “quality of life”, and “depressive condition” were compared between the groups with and without changes in working situations as shown in Figure 5. The scale scores for fatigue (FAS), quality of life (EQ), and depression (SDS) were significantly worse in the long COVID patients who had changes in their working situations. That is, the median scores on the FAS (38 vs. 28), were significantly higher in the group with changes in working situations. The scores on the EQ-5D-5L (0.63 vs. 0.82) and EQ-5D VAS (50 vs. 65) were also significantly decreased and the mean score on the SDS (51 vs. 46) was significantly elevated in the group with changes in working situations.

## 4. Discussion

In the present study, we revealed that more than half of the employed patients with long COVID experienced changes in their working situations. There was an increase in the number of patients with changes in working situations, particularly female patients, during the Omicron-variant phase. The major symptoms related to changes in working situations were fatigue, insomnia, headache, and dyspnea, in which fatigue and insomnia showed significantly higher odds ratios using the logistic analysis. Scores for fatigue and for QOL were worse in patients with changes in working conditions and many of those patients had decreases in their incomes. The results of our study reveal that long COVID has a great influence on employment status as well as mental health mainly in middle-aged patients in Japan.

Our survey, conducted in a Japanese hospital, showed that 54% of patients with long COVID who were employed had changes in their employment conditions. A study conducted in Germany showed that 5% of patients took long-term sick leave due to COVID-19 [27]. A 2-month follow-up study for adult patients with COVID-19 in France showed that 11.2% of the employed patients took sick leave [28]. In a Danish study, approximately 3.3% of the patients took long-term sick leave [29]. That study also showed that female patients between the ages of 50 and 65 years who had pre-existing health conditions such as obesity, chronic lung disease, and fibromyalgia tended to show a higher risk of taking long-term sick leave [29]. The results of studies conducted in various countries have shown that the COVID-19 pandemic has had an effect on the labor market, with a high proportion of patients taking time off from work or taking sick leave due to prolonged symptoms of long COVID.

In our study conducted in Japan, the effects of long COVID on employment were more prominent in women especially in the Omicron phase but were not related to the severity of the acute phase. It is still unclear from this study whether this difference is due to the higher frequency of COVID-19 sequelae among women or to gender differences in employment environments. However, of interest is that the patients with sequelae that affected their employment status had significantly higher frequencies of symptoms of fatigue, insomnia, headache, and dyspnea, which were directly linked to a poor QOL and the progression of depressive status. Taken together, there may also be a gender difference in that the fatigue and headache [11] caused by the aftereffects of COVID-19 seen in women have a greater impact on employment conditions or working hours. In this regard, a British study showed that patients with symptoms such as fatigue and cognitive decline, so-called brain fog, had severely limited prospects for returning to work or finding new jobs [14]. In our Japanese study, among the symptoms related to brain fog, headache was also strongly associated with changes in employment status.

A study in which the prevalence and duration of fatigue in long COVID patients were examined revealed that there was no association between conditions related to the severity of the acute phase of COVID-19, such as hospitalization and oxygen inhalation, and fatigue due to long COVID [30]. It has also been reported that long COVID patients with various symptoms were more likely to have anxiety, depression, or a history of antidepressant use and were also more likely to be female [30]. These reports suggest that the impact of COVID-19 and long COVID on the workforce is varied depending not only on the severity of the acute disease but also on the age, gender, and health status of the individual workers. On the other hand, it is interesting that there are some long COVID symptoms, such as loss of smell/taste and hair loss, that do not seem to have a direct effect on employment status.

A survey with three thousand participants, including participants with confirmed COVID-19 and participants suspected of having COVID-19, that was conducted over a seven-month period showed that about 45% of the participants had to reduce their working hours compared to their working hours before they became sick [7]. Additionally, 22% of the participants in that survey were unable to return to work for various reasons, including sick leave, dismissal, resignation, and failure to find work in a job search [7]. In Japan, a doctor’s certificate is officially required for a long-term leave of absence, and that might be a barrier for long COVID patients to return to work. Research has been conducted to clarify barriers and solutions to returning to work from the patient’s perspective [15]. In a Belgian study, some COVID-19 patients described skeptical reactions from employers and colleagues [15]. The results of that study further indicated the need for much longer and more careful support from the social welfare system to facilitate the return to work and the important role of industrial physicians and healthcare workers in helping patients return to work after prolonged symptoms of COVID-19 [15].

The persistence of symptoms also has a significant impact on employment issues. In a Japanese questionnaire survey with 502 respondents, 32% of the respondents had prolonged symptoms at 6 months after diagnosis and 25% of the respondents had prolonged symptoms even at 18 months after diagnosis [31]. Thus, one and a half years after the onset of COVID-19, approximately one in four people still had some symptoms. In other words, even when the limit for injury and sickness allowance in Japan is reached, there is a possibility that the employee will not be able to return to work [31]. Whether or not the proportion of patients suffering from long COVID decreases over time will be a major factor determining the future impact of long COVID on the employment situation [31].

It was of interest that a worsening of QOL and depression occurred in patients whose employment status was affected in the present study in Japan. It has been reported that a decline in work ability and its impact on income and lifestyle after infection may have further influences on the physical and mental health of the affected individuals [32]. It has also been reported that some patients who experience long COVID are severely limited in their ability to return back to work or to find a new job due to fatigue and a decline in cognitive function [14], and it is unclear how long the fatigue will persist. Since some patients feel strong anxiety regarding the possibility of no recovery from their illness [33], patients with long COVID are in a state of physical and mental stress [14].

In our study, 64% of the patients whose employment was affected had decreased incomes. Another Japanese study has shown that a low annual household income is a risk factor for the development of long COVID in children and adolescent patients [34]. In addition to other lifestyle factors, such as health-related exercise and nutritional status, patients in low-income households are less likely to obtain good advice regarding long COVID, and that may lead to delays in appropriate diagnosis and sufficient treatment [34]. The results of that study indicate that a change in employment status and a decrease in household income not only affects the patients themselves but also has the potential to worsen the aftereffects on their families and children [34].

Our study has several limitations as a retrospective study. First of all, the number of patients was relatively small because the entire dataset was derived from a single university hospital. Second, this was a retrospective study in which data were extracted from medical records. Therefore, it was not possible to establish a corresponding control group. It is also possible that patients did not fully report their employment status or the impact of other factors on their health at the time of their visit to their doctor, which may have led to an underestimation of the results. Third, since the phases of virus variants were determined solely based on the infection periods based on the medical records, the viral phases may not exactly indicate the data to compare the phase-dependent issues. Also, information regarding serum antibody levels against the SARS-CoV-2 virus, in addition to vaccination histories, would be informative for predicting long-term fatigue due to long COVID [35]. Fourth, the quarantine periods during the infection as well as the long COVID condition and the patients’ family status might also have affected the prolongation of the long COVID condition. Fifth, in our future studies, further detailed analysis considering multivariable adjustment with a control group will be necessary to make conclusions of the critical factors.

In conclusion, we revealed that 54% of the patients with long COVID who were employed had changes in their working situations due to prolonged symptoms of general fatigue, sleep disturbance, headache, and/or dyspnea. Through our present Japanese survey, it was further recognized that since changes in working situations result in the worsening of individual levels of QOL, long-term social support as well as mental support are necessary for patients with long COVID.

## Figures and Tables

**Table 1:** Long COVID patients classified by changes in working situations.

Changes in Working Situations	Not Changed (*n* = 250)	Changed (*n* = 295)	*p*-Value
Age, median years (IQR)			
	43 (33–49)	43 (32–51)	0.938 ^(a)^
Gender, *n* (%)			
Male	134 (53.6)	131 (44.4)	* 0.032 ^(b)^
Female	116 (46.4)	164 (55.6)	
BMI, median (IQR)			
	22.9 (20.6–25.8)	23 (20.5–27.2)	0.297 ^(a)^
Smoking habit, *n* (%)			
Yes	92 (36.95)	104 (35.62)	0.748 ^(b)^
No	157 (63.05)	188 (64.38)	
Unknown	1	3	
Severity in the acute phase, *n* (%)			
Mild	215 (86)	275 (93.2)	** *p* < 0.01 ^(b)^
Moderate/Severe	35 (14)	20 (6.8)	
Duration between infection and first visit to the CAC, *n* (%)			
≦90 days	105 (42)	160 (54.2)	** *p* < 0.01 ^(b)^
>90 days	145 (58)	135 (45.8)	
Vaccination history, *n* (%)			
0 to 1 time	93 (38.0)	100 (34.1)	0.356 ^(b)^
2 to 5 times	152 (62.0)	193 (65.9)	
Unknown	5	2	
Changes in income, *n* (%)			
Not decreased	162 (66.4)	105 (36.3)	** *p* < 0.01 ^(b)^
Decreased	82 (33.6)	184 (63.7)	
Unknown	6	6	

The data were analyzed by using ^(a)^ the Mann–Whitney U test or ^(b)^ the chi-squared test. ** *p* < 0.01 and * *p* < 0.05 were regarded as statistically significant.

## Data Availability

When requested, the detailed data will be available via the corresponding author.

## Abbreviations

BMI: body mass index; coronavirus disease 2019 (COVID-19); COVID-19 aftercare clinic (CAC); EQ-5D: Euro QOL 5-dimensions 5-levels score; fatigue assessment scale (FAS); post-COVID-19 condition (PCC); quality of life (QOL); self-rated depression scale (SDS); and VAS: visual analog scale.
